# Redox Pioneer: Professor Hideo Kimura

**DOI:** 10.1089/ars.2018.7618

**Published:** 2019-03-29

**Authors:** David Lefer

**Affiliations:** CV Center of Excellence, Louisiana State University Health Sciences Center, New Orleans, Louisiana.

**Keywords:** redox pioneer, polysulfides, hydrogen sulfide, signaling, nitric oxide

## Abstract

Dr. Hideo Kimura is recognized as a redox pioneer because he has published an article in the field of antioxidant and redox biology that has been cited >1000 times, and 29 articles that have been cited >100 times. Since the first description of hydrogen sulfide (H_2_S) as a toxic gas 300 years ago, most studies have been devoted to its toxicity. In 1996, Dr. Kimura demonstrated a physiological role of H_2_S as a mediator of cognitive function and cystathionine β-synthase as an H_2_S-producing enzyme. In the following year, he showed H_2_S as a vascular smooth muscle relaxant in synergy with nitric oxide and its production by cystathionine γ-lyase in vasculature. Subsequently he reported the cytoprotective effect of H_2_S on neurons against oxidative stress. Since then, studies on H_2_S have unveiled numerous physiological roles such as the regulation of inflammation, cell growth, oxygen sensing, and senescence. He also discovered polysulfides (H_2_S_n_), which have a higher number of sulfur atoms than H_2_S and are one of the active forms of H_2_S, as potent signaling molecules produced by 3-mercaptopyruvate sulfurtransferase. H_2_S_n_ regulate ion channels and transcription factors to upregulate antioxidant genes, tumor suppressors, and protein kinases to, in turn, regulate blood pressure. These findings led to the re-evaluation of other persulfurated molecules such as cysteine persulfide and glutathione persulfide. Dr. Kimura is a pioneer of studies on H_2_S and H_2_S_n_ as signaling molecules.

It is fortunate to come across a secret of nature and pick it up.

—Prof. Hideo Kimura

**Figure:**
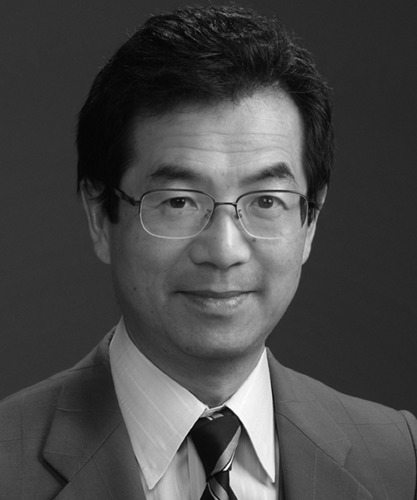
Professor Hideo Kimura

## Background Development and Training

Dr. Kimura graduated from the University of Tokyo, Faculty of Pharmaceutical Sciences, in 1980 and received his PhD from the University of Tokyo in 1985. He studied neurotransmitters in the cerebellum using electrophysiological techniques at the National Defense Medical College (33, 64), and the gene structure of cytochrome P-450 at the Cancer Institute (35, 36). He completed his postdoctoral studies at the Salk Institute for Biological Studies where he identified a novel growth factor, Schwannoma-derived growth factor, as well as activin (28, 32, 34, 71). He continued working at the Salk Institute as a senior staff scientist, where he identified presenilin-binding protein (PBP), a novel guanine nucleotide exchange factor that activates Rac (27). PBP was later renamed as modifier of cell adhesion (9, 60), and finally as dedicator of cytokinases 3 (Dock3) (61).

## Summary of Dr. Kimura's Top Contributions

**FIG. 1.**
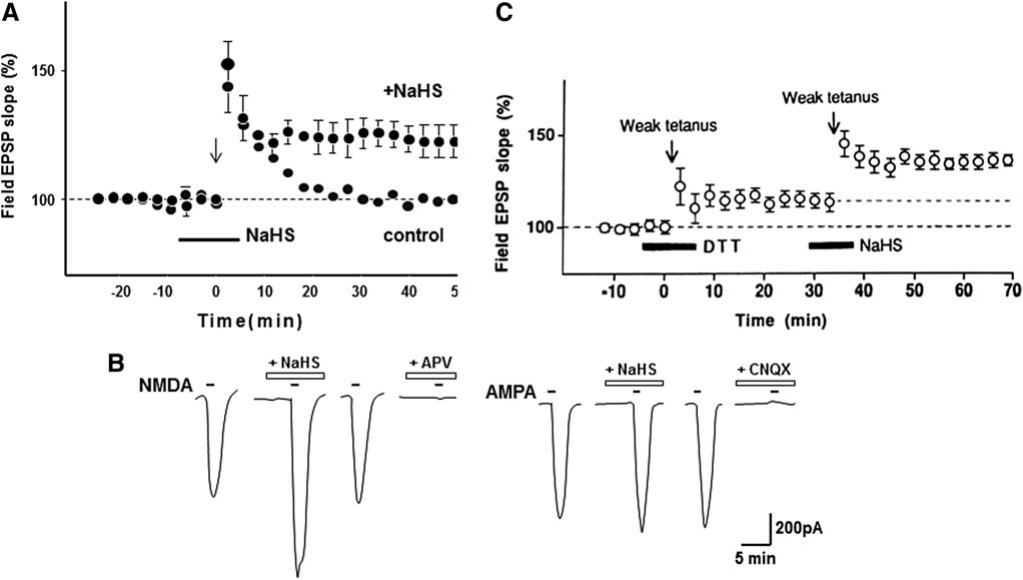
**H_2_S modulates the activity of neurons.** H_2_S facilitates the induction of hippocampal LTP, a synaptic model of memory formation **(A)**, by enhancing the activity of NMDA receptor, leaving another type of glutamate receptor, AMPA receptor, unaffected **(B)** (1). Although a mechanism for the activation of NMDA receptors had been proposed that the reduction of cysteine disulfide bond located at the hinge of the ligand-binding domain by DTT activates the receptor (2), it is not able to fully explain the activation by a weak reducing molecule such as H_2_S. At much lower concentrations, H_2_S (100 μ*M*) induced LTP more efficiently than 1 m*M* DTT **(C)** (1). *Lower dotted line:* control EPSP slope; *upper dotted line:* EPSP slope in the presence of DTT. This finding led to the identification of H_2_S_n_ as novel signaling molecules (40–43, 55, 59, 66) (see Key Finding 3). AMPA, α-amino-3-hydroxy-5-methyl-4-isoxazolepropionic acid; DTT, dithiothreitol; EPSP, excitatory post-synaptic potential; H_2_S, hydrogen sulfide; LTP, long-term potentiation; NMDA, *N*-methyl d-aspartate.

**FIG. 2.**
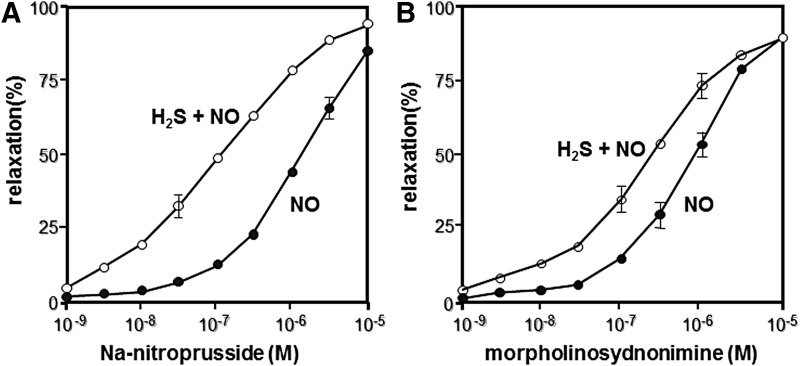
**H_2_S relaxes vascular smooth muscle in synergy with NO.** Synergistic effect of H_2_S and NO was discovered on vascular smooth muscle relaxation. The simultaneous application of H_2_S and NO donors induced greater relaxation of vascular smooth muscle than either H_2_S or NO alone (21). Potentiation of the relaxation effects of Na-nitroprussside **(A)** and morpholinosydnonimine **(B)** by 30 μm NaHS. This finding opened the study of crosstalk between H_2_S and NO and led to the identification of H_2_S_n_ generated by the chemical interaction of both molecules (52) (see Key Finding 3). NO, nitric oxide.

Dr. Kimura showed the first time that hydrogen sulfide (H_2_S) is a novel signaling molecule in multiple body systems. He discovered a physiological role of H_2_S as a mediator of memory formation (1) (Fig. 1), and subsequently identified another role as a vascular smooth muscle relaxant in synergy with nitric oxide (NO) that was the first demonstration of a crosstalk between H_2_S and NO (21) (Fig. 2). Prominent neuroscientist Solomon Snyder commented in *Science News*, “They have very impressive evidence that H_2_S is a potential neurotransmitter. It's an exciting paper that should stimulate a lot of people's interest” (84).

**FIG. 3.**
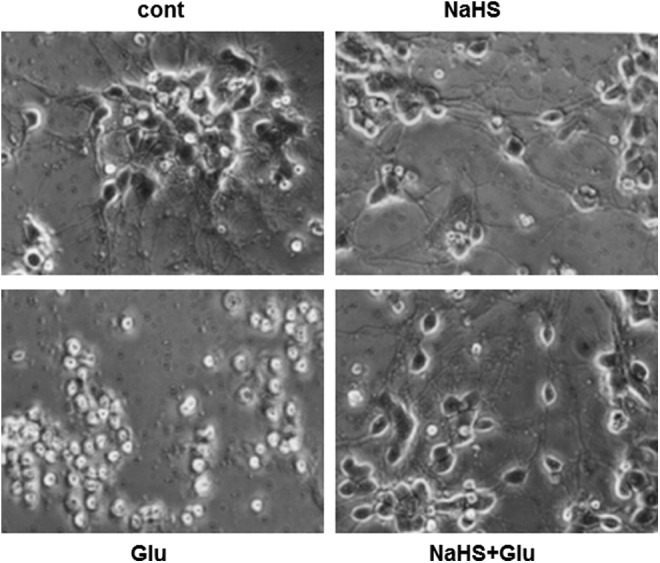
**H_2_S protects neurons from oxidative stress.** Since H_2_S is a well-known toxic gas, its cytoprotective effects have been overlooked. Neurons in primary culture were killed by oxidative stress induced by high concentrations of glutamate, while surviving in the presence of NaHS (39).

Dr. Kimura discovered the cytoprotective effect of H_2_S on neurons from oxidative stress (39) (Fig. 3), and the effect on other tissues and organs such as heart and kidney was followed (14, 85). These findings led to the identification of numerous physiological roles of this molecule, including anti-inflammation, angiogenesis, oxygen sensing, and ATP formation (11, 51, 65, 67, 68, 82, 93, 94).

**FIG. 4.**
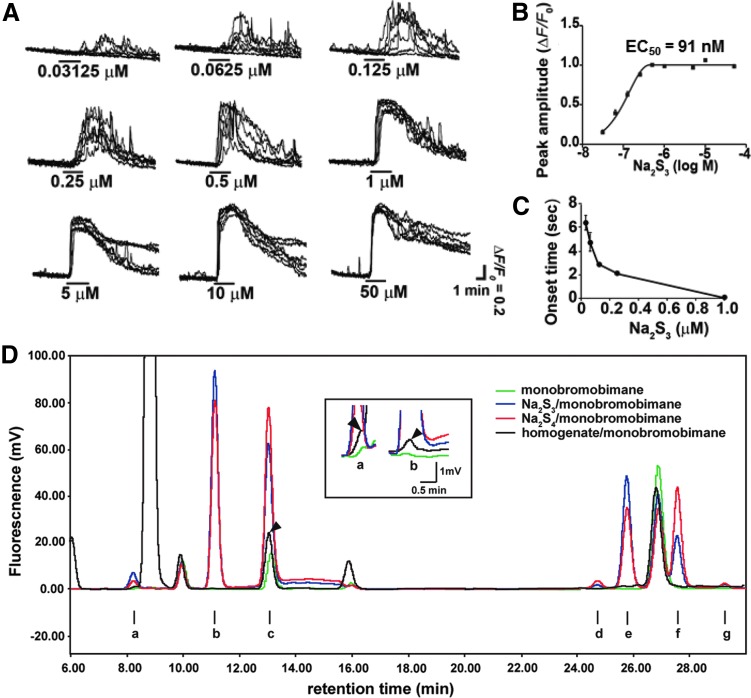
**H_2_S_n_ induce Ca^2+^ influx in astrocytes.** During the study of the effect of H_2_S on astrocytes, Dr. Kimura's group found that a solution of NaHS, a sodium salt of H_2_S whose color is yellowish, activates astrocytes much more efficiently than a colorless solution. The study identified that the yellowish color is derived from H_2_S_n_ generated by the oxidation of H_2_S (40–42, 59, 66). H_2_S_3_ induced Ca^2+^ influx with an EC_50_ value (91 n*M*) **(A, B)**, approximately 1/1000th of that of H_2_S (116 μ*M*) (41). Time required for the induction of Ca^2+^ influx by Na_2_S_3_ **(C)**. Endogenous H_2_S_2_ and H_2_S_3_ were identified in the brain **(D)** (40–43, 55). The enlarged peaks a and b are shown in the *inset*. Major peaks for Na_2_S_3_ and Na_2_S_4_ are b and c, respectively. Since the peaks a, b, and c were observed in a control sample with monobrombimane alone, it was subtracted from each peak of the sample to estimate the amount of polysulfides in brain homogenates.

**FIG. 5.**
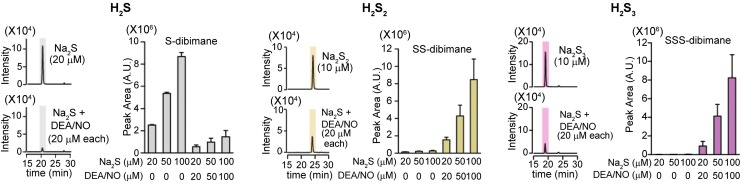
**H_2_S_n_ are generated from H_2_S and NO.** Chemical interaction between H_2_S and NO produces H_2_S_2_ and H_2_S_3_ (52). It may be one of the mechanisms for the synergistic effects of H_2_S and NO on various tissues, including vascular smooth muscle relaxation (12, 21) as shown in Figure 2.

During further investigation of the physiological roles of H_2_S, Dr. Kimura discovered novel signaling molecules, H_2_S_n_, produced by 3-mercaptopyruvate sulfurtransferase (3MST) (41, 42, 58, 59, 66) (Fig. 4). Since then, studies on H_2_S_n_ have unveiled various physiological roles such as the regulation of ion channels, transcription factors, protein kinases, and tumor suppressors, as well as the production of other per- and polysulfurated molecules (18, 41, 44, 80). Dr. Kimura also identified H_2_S_n_ produced by the chemical interaction of H_2_S with NO that may provide one of the mechanisms of the synergy between H_2_S and NO (52) (Fig. 5).

## Description of Key Finding 1

In the library at the Salk Institute, Dr. Kimura found a metabolic map showing mammalian enzymes that can produce H_2_S. These enzymes were intensively studied from the 1950s to 1970s; cystathionine β-synthase (CBS), cystathionine γ-lyase (CSE), and 3MST together with cysteine amino transferase were found to have the capacity *in vitro* (5, 8, 48, 79), and their full-length cDNAs were cloned in the 1990s (56, 62, 81). However, rather than being recognized as a physiologically active molecule in these early studies, H_2_S was thought of simply as a byproduct of metabolic pathways or a marker for the evaluation of enzyme activity.

Three groups discovered endogenous sulfide in mammalian brains when measuring sulfide levels in intoxicated animals (17, 70, 88). Survivors of H_2_S poisoning experienced memory loss, and acute intoxication with H_2_S caused changes in the levels of neurotransmitters in the brains of animal models. Inspired by these findings, Dr. Kimura began studying H_2_S as a signaling molecule in 1993 when carbon monoxide had just been identified as a gaseous signaling molecule (47, 78, 86, 95), following on NO (4, 16, 23).

**FIG. 6.**
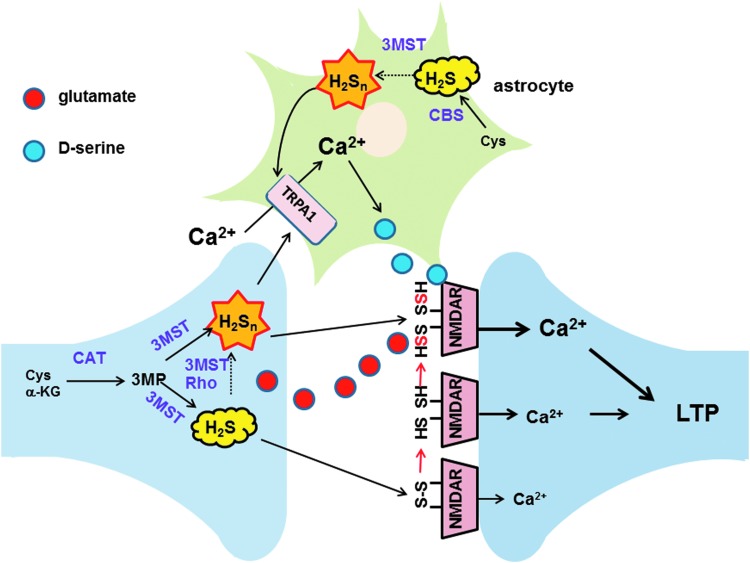
**Facilitation of LTP induction by H_2_S and H_2_S_n_.** H_2_S enhances the activity of NMDA receptors by reducing the cysteine disulfide bond at the hinge of the ligand-binding domain of the receptors (1). H_2_S_n_ activate TRPA1 channels to induce Ca^2+^ influx in astrocytes (41, 59, 66), which, in turn, release gliotransmitters such as d-serine to enhance the activity of NMDA receptors (77). By these integrated mechanisms, LTP may be effectively induced. Modified from Kimura (29). 3MST, 3-mercaptopyruvate sulfurtransferase; CBS, cystathionine β-synthase; TRPA1, transient receptor potential ankyrin 1.

In 1996, Dr. Kimura together with his student Dr. Kazuho Abe demonstrated that H_2_S, which can be produced by CBS in the brain, facilitates the induction of hippocampal long-term potentiation (LTP), a synaptic model of memory formation (1) (Figs. 1 and 6). H_2_S enhances the activity of one type of glutamate receptor, *N*-methyl d-aspartate (NMDA) receptor, leaving another type of α-amino-3-hydroxy-5-methyl-4-isoxazolepropionic acid (AMPA) receptor, which is also activated by glutamate, unaffected. A mechanism for the activation of NMDA receptors had been proposed by Aizenman *et al.* using dithiothreitol (DTT), which reduces cysteine disulfide bond located at the hinge of the ligand-binding domain to activate the receptor (2). However, this mechanism is not able to fully explain the activation by a weak reducing molecule such as H_2_S. At much lower concentrations, H_2_S induced LTP more efficiently than DTT (Fig. 1) (1). This finding led to the identification of H_2_S_n_ as novel signaling molecules (see Key Finding 3).

In 1997, Dr. Kimura demonstrated that H_2_S can be produced by CSE to relax vasculature in synergy with NO (21) (Fig. 2). This discovery also opened a field of crosstalk between H_2_S and NO (see also Key Finding 3). Subsequently, Wang and colleagues identified K_ATP_ channels as one of the targets of H_2_S to relax vascular smooth muscle (94), and Cirino and colleagues showed cyclic GMP-dependent protein kinase as another target to mediate H_2_S-induced vasorelaxation (6).

## Description of Key Finding 2

**FIG. 7.**
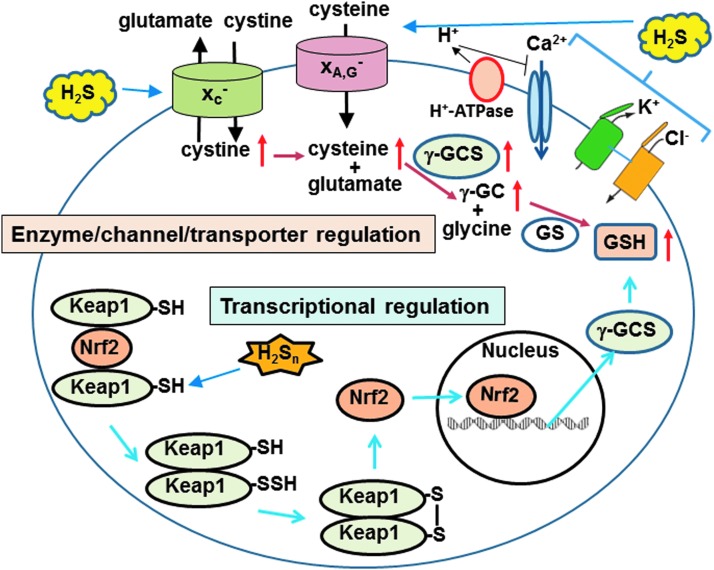
**Protection of neurons from oxidative stress by H_2_S and H_2_S_n_.** H_2_S enhances the activity of the cystine/glutamate antiporter to increase the transport of cystine, which is reduced to cysteine in cells (39), and also enhances the activity of the cysteine transporter (38). H_2_S upregulates the activity of a rate limiting enzyme for GSH production, γ-GCS, also known as GCL. By these effects, H_2_S increases the production of GSH. H_2_S also enhances the activity of K_ATP_ channels and CFTR Cl^−^ channels to suppress excessive excitation by stabilizing the membrane potential (37), while it suppresses the voltage gated Ca^2+^ channels to decrease Ca^2+^ toxicity by enhancing the activity of H^+^-ATPase (49). In contrast, H_2_S_n_ facilitate the release of Nrf2 from Keap1/Nrf2 complex by sulfurating cysteine residues of Keap1, resulting in the transport of Nrf2 to the nucleus where Nrf2 upregulates the transcription of antioxidant genes, including γ-GCS (GCL) (44). By these integrated mechanisms, H_2_S and H_2_S_n_ protect neurons from oxidative stress. γ-GCS, γ-glutamyl cysteine synthetase; GCL, glutamate cysteine ligase; GSH, glutathione; Keap1, kelch-like ECH-associated protein 1; Nrf2, nuclear factor-like 2.

Since H_2_S is a well-known toxic gas, its cytoprotective effects have been overlooked. Dr. Kimura found that H_2_S protects neurons from oxidative stress through enhancing the activity of the cystine/glutamate antiporter and cysteine transporter, as well as γ-glutamyl cysteine synthetase or glutamate cysteine ligase, a rate limiting enzyme in the production of glutathione (GSH), which is a major cellular antioxidant (38, 39) (Figs. 3 and 7). Dr. Kimura also showed that H_2_S enhances the activity of K_ATP_ and CFTR Cl^-^ channels to suppress the excessive excitation of neurons by stabilizing membrane potential (37) (Fig. 7). This finding led to the identification of the cytoprotective effect of H_2_S on various tissues and organs, including the heart, kidney, retina, pancreas, and intestines, and the regulation of endoplasmic reticulum stress (14, 26, 45, 49, 72, 85, 93). Several H_2_S-based therapeutic compounds have been developed, with some undergoing clinical trials (87). H_2_S even plays an essential role in the development of bacterial resistance to antibiotics (73). H_2_S is a universal cytoprotectant effective in bacteria and mammals.

Dr. Kimura's group also identified the transcription factor specific protein 1 (SP1)-binding site at the 5′-noncoding region of the CSE gene (25) that contributes to the cytoprotection *via* NF-κB signaling. Snyder and colleagues identified a mechanism for the antiapoptotic actions induced by tumor necrosis factor alpha (TNFα), where TNFα stimulates the binding of SP1 to the CSE promoter that increases the levels of CSE, resulting in the production of H_2_S. H_2_S S-sulfurates (sulfhydrates) cysteine residues of the p65 subunit of NF-κB to facilitate the interaction with another subunit RPS3, leading to upregulation of antiapoptotic genes (72).

H_2_S-producing pathway from d-cysteine that also contributes to cytoprotection was also identified by Dr. Kimura's group (74). d-Cysteine is metabolized by d-amino acid oxidase (DAO) to 3-mercaptopyruvate (3MP), which is a substrate of 3MST to produce H_2_S (75, 76). In the kidney, H_2_S production from d-cysteine is ∼80 times greater than that from l-cysteine, and the administration of d-cysteine protects the kidney from ischemia-reperfusion injury more efficiently than that of l-cysteine. 3MST is a ubiquitous enzyme, whereas DAO is expressed only in restricted tissues, such as the brain and kidney, in mice. This finding may suggest a new therapeutic approach to deliver H_2_S to specific tissues such as the brain and kidney.

## Description of Key Finding 3

Astrocytes, a type of glia, were thought to merely support and provide nutrients to neurons, but were recently recognized as actively regulating neuronal activity by releasing gliotransmitters such as d-serine to synaptic clefts. Dr. Kimura's group found that H_2_S induces Ca^2+^ influx in astrocytes (58). However, during this study, it was found that a solution of NaHS, a sodium salt of H_2_S whose color is yellowish, activates astrocytes much more efficiently than a colorless solution. The study identified that the yellowish color is derived from H_2_S_n_ generated by the oxidation of H_2_S, and it measured the endogenous H_2_S_n_ in the brain (41, 42, 59, 66) (Fig. 4).

H_2_S_3_ induced Ca^2+^ influx with an EC_50_ value (91 n*M*), approximately 1/1000th of that of H_2_S (116 μ*M*) (Fig. 4). The effect of H_2_S_3_ was suppressed by inhibitors as well as siRNAs specific to transient receptor potential ankyrin 1 (TRPA1) channels, suggesting that H_2_S_3_ activates TRPA1 channels (41). In collaboration with Dr. Ohta, Dr. Kimura identified the target of H_2_S_3_ as two cysteine residues located at the amino terminus of TRPA1 channels (20). This finding suggests the additional mechanism for the facilitation of LTP induction (see Key Finding 1, and Fig. 6). H_2_S_n_ activates TRPA1 channels to induce Ca^2+^ influx in astrocytes (41, 59, 66), which, in turn, release gliotransmitters such as d-serine to enhance the activity of NMDA receptors (29, 77).

Other roles of H_2_S_n_ were subsequently found (30). In collaboration with Dr. Ogasawara, Dr. Kimura found that H_2_S_n_ facilitate the translocation of nuclear factor-like 2 (Nrf2) to the nucleus by modifying its binding partner kelch-like ECH-associated protein 1 (Keap1) to upregulate the transcription of antioxidant genes (44) (Fig. 7). This system was initially reported to be activated by H_2_S (7). H_2_S_n_ regulate the activity of the tumor suppressor phosphatase and tensin homolog (18) and reduce blood pressure by dilating vascular smooth muscle through the activation of protein kinase G1α (80). These studies facilitated the identification of the production pathways for H_2_S_n_.

Hylin and Wood reported that persulfurated cysteine residues of proteins were produced from 3MP, a substrate of 3MST (22). Bound sulfane sulfur, which is defined as the sulfur species that releases H_2_S under reducing conditions, includes H_2_S_n_, free cysteine persulfide (Cys-SSH), GSH persulfide (GSSH), and persulfurated cysteine residues of proteins (24, 54, 63, 89). Dr. Kimura's group found that cells expressing 3MST contain higher levels of bound sulfane sulfur than cells expressing defective mutants of 3MST as well as cells without 3MST (76). Oral administration of d-cysteine to mice increases the levels of bound sulfane sulfur in the kidney to which DAO is highly localized (74). As predicted, brains of 3MST knockout mice contain less bound sulfane sulfur than those of wild-type mice (40). These observations suggested that 3MST can also produce a free form of persulfurated molecules such as H_2_S_n_. In collaboration with Dr. Ogasawara and Dr. Nagahara, Dr. Kimura found that 3MST produces H_2_S_2_ and H_2_S_3_ as well as H_2_S, and determined the endogenous levels of both molecules (40, 42, 43, 55).

**FIG. 8.**
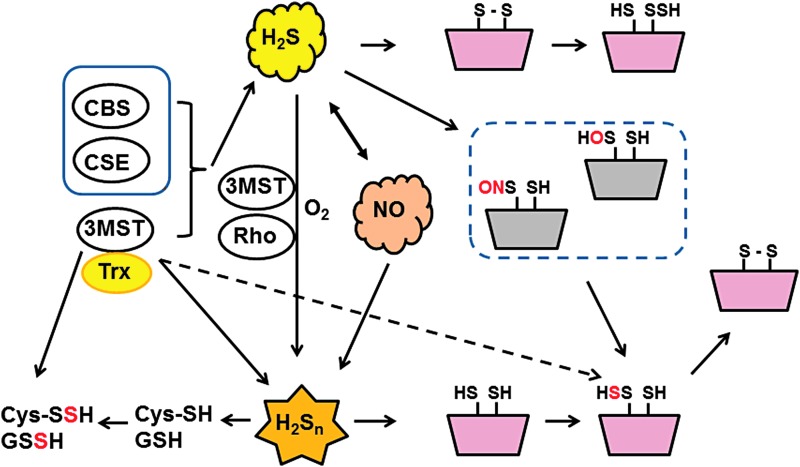
**Production of H_2_S, H_2_S_n_, Cys-SSH, GSSH, and S-sulfurated proteins by 3MST and the effects of these molecules.** 3MST produces H_2_S_n_ and H_2_S by interacting with thioredoxin (Trx) (40–42, 50). H_2_S_n_ are also generated by the interaction of H_2_S with NO or the oxidation of H_2_S (52). 3MST can also produce Cys-SSH and GSSH directly or through the generation of H_2_S_n_, which readily react with Cys-SH and GSH (40). H_2_S reduces the cysteine disulfide bond to induce the conformational changes of target proteins, resulting in the regulation of their activity (1). H_2_S_n_ S-sulfurate (sulfhydrate) cysteine residues of target proteins (54), whereas under oxidative stress or by NO signaling (*dotted box*) H_2_S S-sulfurates the oxidized cysteine residues such as nitrosylated (Cys-SNO) and sulfenic acid (Cys-SOH) to regulate the activity of targets. (Reprinting from Ref. 31). CSE, cystathionine γ-lyase.

During studies on H_2_S_n_ production by 3MST, Dr. Kimura's group noticed that levels of cysteine and GSH were decreased when 3MST produces H_2_S_n_. Dr. Kimura concluded that H_2_S_n_ readily reacted with Cys-SH and GSH to produce Cys-SSH and GSSH (40). Alternatively, 3MST may transfer sulfur from 3MP to cysteine and GSH to produce these persulfurated species (Fig. 8).

3MST requires a reducing substance to produce H_2_S, but an endogenous reducing substance was not known. From the structure of leishmania 3MST, which contains a thioredoxin domain in the molecule, the interaction of thioredoxin with 3MST had been predicted (90). Dr. Kimura's group determined thioredoxin as well as dihydrolipoic acid as potential endogenous reducing molecules necessary for 3MST to produce H_2_S (50, 57, 92).

3MST thiolates tRNA to maintain the accuracy of the genetic code and stabilize the tRNA structure (46, 91). There are two isoforms of 3MST that localize to the cytosol and mitochondria in humans (15). Cytosolic isoforms thiolate tRNA, whereas the mitochondrial isoform has a dual localization in both mitochondria and the cytosol, and not only thiolates tRNA in the cytosol but also supplies sulfur for iron–sulfur cluster formation in mitochondria (15). It is interesting to note that cysteinyl-tRNA synthetase was recently reported to have the activity to produce Cys-SSH (3).

Eberhardt *et al.* and Cortese-Krott *et al.* reported that the chemical interaction of H_2_S with NO produces HNO and SSNO^−^, respectively (10, 13). Both groups showed H_2_S_n_ formation, but they did not consider their physiological relevance. Whereas Eberhardt *et al.* concluded that HNO is the chemical species responsible for activating the TRPA1-CGRP neuroendocrine signaling cascade and postulated that this pathway is essential for control of the general vascular tone, Dr. Kimura's group determined that H_2_S_2_ and H_2_S_3_ are the molecules that activate the TRPA1 channels of dorsal root ganglion neurons (52) (Fig. 5). Mustafa and Habara reported that H_2_S_n_ must be produced by the interaction of H_2_S and NO in mast cells (53). Molecules, which are produced from H_2_S and NO and activate TRPA1 channels, and H_2_S_n_ are degraded by cyanide (cyanolysis) and by reduction (52). In contrast, HNO is resistant to cyanolysis and SSNO^−^ to reduction (10, 13). Considering these observations, Kimura's group suggested that the production of H_2_S_n_ from H_2_S and NO may be one of the mechanisms for the synergistic effects of both molecules on various tissues, including vascular smooth muscle relaxation (12, 21).

## Other Achievements

In collaboration with Dr. Nagano, Dr. Urano, and Dr. Hanaoka, Dr. Kimura's group contributed to the development of H_2_S- and polysulfide-fluorescence probes (52, 69, 83) as well as inhibitors specific to 3MST (19).

## Current Position

Dr. Kimura is a specially appointed researcher, National Institute of Neuroscience, National Center of Neurology and Psychiatry. He was recently appointed as Professor and Faculty of Pharmaceutical Science at the Tokyo University of Science, Yamaguchi (renamed Sanyo-Onoda City University in 2018), Japan.

He received the Promoting Award from the Japanese Pharmacological Society (1988), Human Frontier Science Program (1990), First Award from National Institute of Health (1994), Alzheimer Scholar Award from Alzheimer Association (1994), JB Award from Japanese Biological Society (2010), Research Front Award from Thomson Reuters (2016), Highly Cited Researcher from Clarivate Analytics (2017), and President Award from the National Institute of Neuroscience, National Center of Neurology and Psychiatry (2018). He has served as a nominator of Japan Prize since 2008.

## Supplementary Material

Supplemental data

Supplemental data

## Abbreviations Used

3MP: 3-mercaptopyruvate
3MST: 3-mercaptopyruvate sulfurtransferase
AMPA: α-amino-3-hydroxy-5-methyl-4-isoxazolepropionic acid
CBS: cystathionine β-synthase
CSE: cystathionine γ-lyase
DAO: d-amino acid oxidase
Dock3: dedicator of cytokinases 3
DTT: dithiothreitol
GSH: glutathione
H_2_S: hydrogen sulfide
Keap1: kelch-like ECH-associated protein 1
LTP: long-term potentiation
NMDA: *N*-methyl d-aspartate
NO: nitric oxide
Nrf2: nuclear factor-like 2
PBP: presenilin-binding protein
SP1: specific protein 1
TNFα: tumor necrosis factor alpha
TRPA1: transient receptor potential ankyrin 1
