# Supplementary material for: Redox Pioneer: Professor Hideo Kimura
Source: Antioxid Redox Signal. 2019 Mar 29;30(14):1699–708. doi: 10.1089/ars.2018.7618 (PMC6477590; doi:10.1089/ars.2018.7618)
Supplement: Supplemental data [file Supp_Table1.pdf]

Supplementary Data

SUPPLEMENTARY TABLE S1. ARTICLE PUBLISHED BY DR. HIDEO KIMURA CITED AT LEAST 1000 TIMES

Publication	Total citations as of 10/05/18
Abe, K. and Kimura, H.: The possible role of hydrogen sulfide as an endogenous neuromodulator. *J. Neurosci.* 16: 1066-1071, 1996.	1159

This citation number was obtained through the Clarivate Analytics Web of Science® database
