# Supplementary material for: Redox Pioneer: Professor Hideo Kimura
Source: Antioxid Redox Signal. 2019 Mar 29;30(14):1699–708. doi: 10.1089/ars.2018.7618 (PMC6477590; doi:10.1089/ars.2018.7618)
Supplement: Supplemental data [file Supp_Table2.pdf]

SUPPLEMENTARY TABLE S2. ARTICLE PUBLISHED BY DR. HIDEO KIMURA CITED AT LEAST 100 TIMES

	Publication	Total citations as of 10/05/18
1.	Abe, K. & Kimura, H.: The possible role of hydrogen sulfide as an endogenous neuromodulator. J. Neuroscience. 16: 1066-1071, 1996.	1159
2.	Hosoki, R., Matsuki, N. & Kimura, H. The possible role of hydrogen sulfide as an endogenous smooth muscle relaxant in synergy with nitric oxide. *Biochem. Biophys. Res. Comm.* 237: 527-531, 1997.	756
3.	Liu, Y, Peterson, D.A., Kimura, H., & Schubert, D. Mechanism of cellular 3-(4,5-dimethylthiazol-2-yl)-2,5-diphenyltetrazolium bromide (MTT) reduction. *J. Neurochem.* 69: 581-593, 1997.	623
4.	Elrod, JW., Calvert, JW., Morrison, J., Doeller, JE, Kraus, DW., Tao, L., Jiao, X., Scalia, R., Kiss, L., Szabo, C., Kimura, H., Chow, CW., Lefer, DJ. Hydrogen sulfide attenuates myocardial ischemia-reperfusion injury by preservation of mitochondrial function. *Proc. Natl. Acad. Sci. USA.* **104:** 11560-11565, 2007.	618
5.	Kimura, Y. and Kimura, H. Hydrogen sulfide protects neurons from oxidative stress. *FASEB J.* 18: 1165-1167, 2004.	504
6.	Shibuya, N., Tanaka, M, Yoshida, M., Ogasawara, Y., Togawa, T., Ishii, K. and Kimura, H. 3-Mercaptopyyruvate sulfurtransferase produces hydrogen sulfide and bound sulfane sulfur in the brain. *Antioxid. Redox Signal.* 11: 703-714, 2009.	463
7.	Sasakura, K., Hanaoka, K., Shibuya, N., Mikami,Y., Kimura, Y., Komatsu, T., Ueno, T., Terai, T., Kimura, H., and Nagano, T. Development of a highly selective fluorescence probe for hydrogen sulfide. *J. Am. Chem. Soc.* 133: 18003-18005, 2011.	377
8.	Schubert, D., Behl, C., Lesley, R., Brack, A., Dargusch, R., Sagara, Y., & Kimura, H.: Amyloid peptides are toxic via a common oxidative mechanism. *Proc. Natl. Acad. Sci. USA.* 92: 1989-1993, 1995.	316
9.	Kimura, H. Hydrogen sulfide: its production, release and functions. *Amino Acids.* 41: 113-121, 2011.	299
10.	Kimura, Y., Goto, Y., and Kimura, H. Hydrogen sulfide increases glutathione production and suppresses oxidative stress in mitochondria. *Antioxid. Redox Signal.* 12: 1-13, 2010.	295
11.	Schubert, D., Kimura, H., LaCorbiere, M., Karr, D., Vaughn, J., & Fischer, W.: Activin is a nerve cell survival protein. *Nature* 344: 868-870, 1990.	295
12.	Kimura, H. Hydrogen sulfide as a neuromodulator. *Mol. Neurobiol.* 26: 13-19, 2002.	273
13.	Ishigami, M., Hiraki, K., Umemura, K., Ogasawara, Y., Ishii, K. and Kimura, H. A source of hydrogen sulfide and a mechanism of its release in the brain. *Antioxid. Redox Signal.* 11: 205-214, 2009.	272
14.	Shibuya, N., Mikami, Y., Kimura, Y., Nagahara, N., and Kimura, H. Vascular endothelium expresses 3-mercaptopyruvate sulfurtransferase and produces hydrogen sulfide. *J. Biochem.* 146: 623-626, 2009.	257
15.	Kimura, H. Hydrogen sulfide induces cyclic AMP and modulates the NMDA receptor. *Biochem. Biophys. Res. Commun.* 267: 129-133, 2000.	239
16.	Nagai, Y., Tsugane, M., Oka, J. and Kimura, H. Hydrogen sulfide induces calcium waves in astrocytes. *FASEB J.* 18: 557-559, 2004.	202
17.	Kimura, Y. Dargusch, R. Schubert, D. and Kimura, H. Hydrogen Sulfide Protects HT22 Neuronal Cells from Oxidative Stress. *Antioxid. Redox Signal.* **8:** 661-670, 2006.	191
18.	Ishii, I., Akahoshi, N., Yu, X., Kobayashi, Y., Namekata, K., Komaki, G. and Kimura, H. Murine cystathionine gamma-lyase: complete cDNA and genomic sequences, promoter activity, tissue distribution, and developmental expression. *Biochem. J.* 381: 113-123, 2004.	178
19.	Kimura, H. Hydrogen sulfide: from brain to gut. *Antioxid. Redox Signal.* 12: 1111-1123, 2010.	178
20.	Shibuya, N., Koike, S., Tanaka, M., Ishigami-Yuasa, M., Kimura, Y., Ogasawara, Y., Fukui, K., Nagahara, N. and Kimura, H. A novel pathway for the production of hydrogen sulfide from D-cysteine in mammalian cells. *Nature Commun.* 4: 1366, 2013.	161
21.	Schicho, R., Krueger, D., Zeller, F., Weyhern, C.W.H.V, Frieling, T., Kimura, H., Ishii, I., Giorgio, R.D., Campi, B. and Schemann, M. Hydrogen sulfide is a novel prosecretory neuromodulator in the guineapig and human colon. *Gastroenterology* 131: 1542-1552, 2006.	157
22.	Kaneko, Y., Kimura, Y., Kimura, H. and Niki, I. L-cysteine inhibits insulin release from the pancreatic β-cell. Possible involvement of metabolic production of hydrogen sulfide, a novel gasotransmitter. *Diabetes* **55:** 1391-1397, 2006.	155
23.	Schubert, D., Kimura, H., & Maher, P.: Growth factors and Vitamin E modify neuronal glutamate toxicity. *Proc. Natl. Acad. Sci. USA.* 89: 8264-8267, 1992.	154
24.	Kimura, H., Shibuya, N., Kimura, Y. Hydrogen sulfide is a signaling molecule and a cytoprotectant. *Antioxid. Redox Signal.* 17: 45-57, 2012.	152
25.	Kimura, Y., Mikami, Y., Osumi, K., Tsugane, M., Oka, J-I, and Kimura, H. Polysulfides are possible H_2S-derived signaling molecules in rat brain. *FASEB J.* 27: 2451-2457, 2013.	147
26.	Kimura, H., Fischer, W.H., & Schubert. D.: Structure, expression and function of a schwannoma-derived growth factor. *Nature* 348: 257-260, 1990.	142
27.	Kimura, H., Nagai, Y., Umemura, K. and Kimura, Y. Physiological roles of hydrogen sulfide. Synaptic modulation, neuroprotection and smooth muscle relaxation. *Antioxid. Redox Signal.* 7: 795-803, 2005.	137
28.	Mikami, Y. Shibuya, N., Kimura, Y., Ogasawara, Y., and Kimura, H. Thioredoxin and dihydrolipoic acid are endogenous reductants required for 3-mercaptopyruvate sulfurtransferase to produce hydrogen sulfide. *Biochem. J.* 439: 479-485, 2011.	119
29.	Kimura, H. The physiological role of hydrogen sulfide and beyond. *Nitric Oxide* 41: 4-10, 2014.	106

This citation number was obtained through the Clarivate Analytics Web of Science® database
